# Chemical Composition, Antioxidant, and Enzyme Inhibitory Activities of *Artemisia schmidtiana* Maxim. Essential Oil

**DOI:** 10.3390/biom15050736

**Published:** 2025-05-19

**Authors:** Xinyu Zhu, Xu Liu

**Affiliations:** 1 SDU-ANU Joint Science College, Shandong University, Weihai 264209, China; 202200700229@mail.sdu.edu.cn; 2 Marine College, Shandong University, Weihai 264209, China

**Keywords:** *Artemisia schmidtiana* Maxim., essential oil, chemical composition, biological activity, molecular docking

## Abstract

*Artemisia schmidtiana* Maxim., a plant belonging to the Asteraceae family, is renowned for its extensive ethnomedicinal applications and distinctive aromatic qualities. This study evaluated the chemical composition, antioxidant capacity, and inhibitory effects on acetylcholinesterase (AChE), α-glucosidase, and β-lactamase of its essential oil (EO). The major constituents of the EO were identified as germacrene D (16.29%), falcarinol (11.02%), β-caryophyllene (9.43%), α-zingiberene (7.93%), phytol (6.06%), and α-humulene (4.04%). The EO demonstrated radical scavenging activity against DPPH (44.9% at 5 mg/mL) and ABTS (IC_50_ = 0.72 ± 0.02 mg/mL) radicals, with a FRAP antioxidant capacity of 126.61 ± 0.59 μmol·g^−1^. Additionally, the EO exhibited modest AChE inhibition (16.7% at 250 μg/mL) and significant inhibition of α-glucosidase and β-lactamase, with IC_50_ values of 178.80 ± 17.02 μg/mL and 40.06 ± 8.22 μg/mL, respectively. Molecular docking revealed favorable interactions between the major EO compounds and the tested enzymes, providing a theoretical foundation for future drug development. These findings suggest that *A. schmidtiana* EO holds potential for applications in the food and pharmaceutical industries, warranting further investigation.

## 1. Introduction

Plants represent one of the primary sources of raw materials for the food, pharmaceutical, and cosmetic industries, with many nutraceuticals and cosmeceuticals deriving their medicinal value from plant-based constituents [1]. Among various plant-derived products, essential oil (EO), a complex mixture of hydrophobic volatile compounds originating from plant secondary metabolism, is a promising molecular library for drug discovery [2]. This oil exhibits specific bioactivities, including antimicrobial, anti-inflammatory, and antioxidant properties [3,4]. Furthermore, the emergence of green consumerism has increased consumer demand for natural-extracted pharmaceutical agents with minimal environmental footprints. As natural products, EO has been widely utilized in traditional medicine to promote human health and treat diseases, owing to its intriguing biochemical profiles and high added value in environmental sustainability [5].

In exploring the bioactivity of EO, its antioxidant capacity is often regarded as paramount, given the central role of oxidative stress in various pathological processes [6]. Oxidative stress arises from either excessive production of free radicals or compromised antioxidant defense systems [7,8]. Free radicals, characterized by unpaired electrons, are highly reactive and readily interact with cellular components [9]. While they participate in critical cellular processes, an overabundance of free radicals can inflict widespread damage on cellular structures [1]. This damage is implicated in the pathogenesis of numerous chronic and degenerative diseases, including Alzheimer’s disease (AD), diabetes mellitus (DM), and cardiovascular disorders [10]. Nevertheless, this detrimental process can be modulated through exogenous antioxidants derived from natural sources, such as plant EO and extracts, which offer a promising therapeutic avenue [11].

AD is an age-related neurodegenerative disorder characterized by three hallmark features: the deposition of amyloid-β (Aβ) plaques, hyperphosphorylation of the cytoskeletal protein tau, and reduced levels of acetylcholine (ACh), leading to neuronal dysfunction [12]. Among these, Aβ accumulation plays a pivotal role in driving oxidative stress [13]. Concurrently, AD patients exhibit decreased levels of antioxidants, such as uric acid, vitamin C, and vitamin E, and reduced activity of antioxidant enzymes like superoxide dismutase and catalase [14]. These findings underscore oxidative stress as a key contributor to AD pathogenesis, suggesting that antioxidant supplementation may offer therapeutic benefits [15]. Furthermore, acetylcholinesterase (AChE), a serine protease that hydrolyzes ACh in the brain, contributes to AD progression by diminishing ACh levels and disrupting neuronal signaling [16]. Notably, EO can cross the blood–brain barrier and modulate the central nervous system, potentially alleviating AD symptoms due to its small molecular size and lipophilic properties [17]. Therefore, EO is a promising candidate for developing novel therapeutics targeting neurodegenerative diseases.

DM is a chronic disease characterized by elevated blood glucose levels due to insulin deficiency or impaired insulin function [18]. Oxidative stress is widely recognized as a pivotal contributor to the pathogenesis of DM, primarily through mechanisms such as mitochondrial H_2_O_2_ production and NADPH oxidase activation, which disrupt insulin signaling and promote insulin resistance [19]. Consequently, antioxidants demonstrate beneficial effects in DM treatment. In addition, the inhibition of α-glucosidase, a carbohydrate-hydrolyzing enzyme located on the intestinal brush border, represents a critical therapeutic strategy. By delaying the digestion of oligosaccharides into absorbable monosaccharides, α-glucosidase inhibitors effectively modulate postprandial glucose absorption, thereby mitigating glycemic fluctuations and slowing disease progression [20]. Recent studies have identified EO derived from 20 plant species with significant potential for DM management, attributed to their dual antioxidant and α-glucosidase inhibitory activities [21]. EO has already been utilized in therapeutic formulations and has demonstrated a favorable safety profile with no significant adverse effects reported [22]. Consequently, EO may offer a promising avenue for discovering and developing novel antidiabetic agents.

β-Lactam antibiotics are widely regarded as one of the most effective antibacterial agents due to their broad spectrum of activity, favorable pharmacokinetics, and safety profile [23]. However, the extensive use of β-lactams has led to the emergence and dissemination of resistance, mediated through diverse mechanisms, including target modification, downregulation of porins required for β-lactam entry, overexpression of efflux systems, and enzymatic modification or degradation [24]. Among these, enzyme-mediated resistance, driven by β-lactamase activity, poses a significant threat. β-Lactamases, produced by both Gram-positive and Gram-negative bacteria, hydrolyze β-lactam antibiotics, rendering one of the most significant threats to antibacterial efficacy [24,25]. In our previous work, *Spermacoce alata* EO exhibited potent β-lactamase inhibitory activity [26]. Therefore, developing EO-based adjunctive therapies may represent a promising strategy to counteract β-lactam resistance and enhance the efficacy of existing antibiotics [27].

*Artemisia*, one of the largest and most widely distributed genera within the Asteraceae family, comprises over 500 species predominantly found in temperate regions of Europe, Asia, and North America [28]. *Artemisia* species are renowned for their diverse bioactivities, including antimicrobial, anticancer, anti-inflammatory, antioxidant, and antipyretic properties, making them highly valued in ethnopharmacology [29,30]. The 2015 Nobel Prize in Medicine, awarded for the discovery of artemisinin, a potent antimalarial sesquiterpene lactone isolated from *Artemisia annua*, renewed scientific focus on these plants, including *Artemisia schmidtiana* Maxim. [30]. *A. schmidtiana*, a perennial herbaceous plant native to alpine or rocky environments, is characterized by its slender, silver–white hairy stems and leaves, reaching heights of approximately 10 cm, and produces small white flowers in July and August. It has historically been prized for its medicinal properties and distinctive aromatic qualities, and it is commonly cultivated as an ornamental plant in gardens and pots, favored by horticultural enthusiasts for its unique foliage. Beyond its traditional uses, thiophene acetylenes were isolated from *A. schmidtiana* in 1986 [31]. Thiophenes, a class of secondary metabolites, exhibit a broad spectrum of biological activities, including antimicrobial, antiviral, HIV-1 protease inhibitory, and anticancer effects, highlighting the plant’s potential for further pharmaceutical development [32].

The previous literature has documented the broad bioactivities and high medicinal value of *A. schmidtiana*. However, the chemical composition and biological activities of its EO remain unexplored. To address this gap, we investigated the chemical profile, antioxidant activity, and inhibitory effects of *A. schmidtiana* EO on AChE, α-glucosidase, and β-lactamase. Additionally, molecular docking was employed to elucidate the potential mechanisms underlying its enzyme-inhibitory properties.

## 2. Materials and Methods

### 2.1. Plant Material

Plant samples of aerial parts at the vegetative stage were collected from a commercial nursery in Shanghai City, China (31.2304° N, 121.4737° E) in September 2023. The species was identified as *A. schmidtiana* by Pro. Zhao Hong, through morphological characteristics. The voucher specimen is stored at the Center for Bioscience Analysis and Testing, Shandong University, Weihai, China, with registration number EO2325. The plant samples were refrigerated at −18 °C until EO extraction.

### 2.2. EO Extraction

Fresh leaves and stems (140 g) of the plant material were finely crushed and placed into a 5 L round-bottom flask, and then 3 L of ultrapure water. Hydrodistillation was carried out for approximately 4 h using a Clevenger-type apparatus to isolate the EO from the plant material. The EO was then separated from the aqueous layer using diethyl ether. Subsequently, the extracted EO was dried using sodium sulfate and concentrated using a Termovap sample concentrator (MD200-1, Shanghai Huyi Technology Co., Ltd., Shanghai, China). The obtained EO was stored at a low temperature (4 °C) for further analysis.

### 2.3. GC-MS and GC-FID Analyses

The composition and relative content of the EO were analyzed using gas chromatography–mass spectrometry (GC-MS) and gas chromatography with flame ionization detection (GC-FID). GC-MS analysis was conducted on an Agilent 7890–5975C system (Santa Clara, CA, USA) with an HP-5MS fused silica capillary column (30 m × 0.25 mm, 0.25 μm film thickness, Agilent, Santa Clara, CA, USA). The injector and interface temperatures were maintained at 260 °C and 280 °C, respectively. The oven temperature program began at 50 °C for 4 min, followed by a gradient increase to 280 °C at 6 °C/min, and held for 3 min. Ultra-pure helium (99.999%) served as the carrier gas at a 1.0 mL/min flow rate. The mass spectrometer operated in electron ionization (EI) mode at 70 eV, with a scan range of 25–500 amu and a quadrupole temperature of 150 °C. Samples were prepared as a 1% (*w*/*v*) solution in dichloromethane, and 0.3 µL was injected in splitless mode. A PerkinElmer Clarus 500 system (Shelton, CT, USA) with an HP-5 column (30 m × 0.25 mm, 0.25 μm film thickness, Agilent, Santa Clara, CA, USA) was used for GC-FID analysis. The injector and detector temperatures were set at 260 °C and 305 °C, respectively. The oven temperature followed the same program as GC-MS, with nitrogen as the carrier gas at 1.1 mL/min.

Compound identification was achieved by comparing mass spectra and retention indices (RI) with the NIST20 library and published data. RIs were calculated using GC-MS data from a series of *n*-alkanes (C_8_–C_30_) analyzed under identical conditions.

### 2.4. Antioxidant Activities Evaluation

#### 2.4.1. DPPH Method

The experimental protocol was slightly modified from previously established methods [33]. 6-Hydroxy-2,5,7,8-tetramethylchroman-2-carboxylic acid (Trolox) was used as the positive control. Trolox and the EO stock solution concentrations were prepared in ethanol at 0.5 mg/mL and 50 mg/mL, respectively. Aliquots of 50 μL from either the Trolox or gradient-diluted EO solutions were transferred into a 96-well microplate, followed by 200 μL of 0.17 mmol/L 2,2-diphenyl-1-picrylhydrazyl (DPPH) solution. The microplate was shielded from light and incubated at 25 °C for 30 min. Absorbance was subsequently measured at 516 nm using an Epoch microplate spectrophotometer (BioTek Instruments, Minneapolis, MN, USA). The DPPH radical scavenging capacity (RSC%) was determined using Equation (1):

(1) $$RSC\%=\left(1-\frac{A_{Sample}-A_{Sample\;Blank}}{A_{control}}\right)\times 100\%$$

where A_Sample_ is the absorbance of the tested sample at different concentrations, A_Control_ is the absorbance of the control (ethanolic DPPH solution), and A_Sample Blank_ is the absorbance of the ethanol sample without DPPH.

#### 2.4.2. ABTS Method

The experimental protocol was modified slightly based on established methodologies from prior studies [26]. The ABTS^•+^ radical solution was prepared by combining 7.4 mmol/L 2,2-azino-bis(3-ethylbenzothiazoline-6-sulfonic acid) (ABTS) with 2.6 mmol/L potassium persulfate. The mixture was incubated in the dark at room temperature for 12 h to ensure complete radical generation. Subsequently, 200 μL of the diluted ABTS^•+^ solution was mixed with 50 μL of gradient-diluted ethanolic EO solutions in a 96-well plate. Absorbance was measured at 734 nm six minutes after initiating the reaction. Ethanol served as the blank and dilution solvent, while 0.5 mg/mL Trolox was used as the positive control. The ABTS radical scavenging capacity (RSC%) was calculated to evaluate antioxidant activity using Equation (2):

(2) $$RSC\%=\left(\frac{A_{0}-A}{A_{0}}\right)\times 100\%$$

where A_0_ and A are the absorbance of 200 μL diluted ABTS^•+^ solution mixed with 50 μL ethanol and 50 μL sample solution, respectively, at 734 nm. IC_50_ was then calculated.

#### 2.4.3. Ferric-Reducing Antioxidant Power (FRAP) Method

The assay was performed following established protocols with minor adaptations. Three stock solutions—(i) acetate buffer (pH 3.6), (ii) 10 mM 2,4,6-tripyridyl-s-triazine (TPTZ), and (iii) 20 mM Fe^3+^ solution—were mixed in a 10:1:1 ratio and diluted 50-fold with ethanol to prepare the FRAP working solution. A standard curve was constructed using Trolox. EO samples were serially diluted to 5000, 2500, 1000, 500, 250, 100, 50, and 25 μg/mL concentrations. The EO samples were combined with the FRAP working solution and incubated at 37 °C in the dark. Absorbance was measured at 593 nm, and the antioxidant capacity was expressed as Trolox equivalent antioxidant concentration (TEAC) by referencing the standard curve. All experiments were conducted in triplicate, and results were reported as mean values.

### 2.5. Anti-AChE Activity Test

This assay was conducted using the previously described method with minor modifications [34]. In total, 145 μL of 0.1 mM phosphate buffered saline (PBS) (pH 8.0), 20 μL of ethanolic EO solution, and 15 μL of AChE solution (0.28 U/mL) were combined in a microplate, with galantamine as the positive control. The mixture was incubated at 4.0 °C for 20 min. Subsequently, 10 μL of 15 mM acetylthiocholine iodide (ATCI) and 10 μL of 2 mM 5,5′-dithiobis-(2-nitrobenzoic acid) (DTNB) were added, followed by homogenization for 60 s. Absorbance was measured at 412 nm at 60 s intervals for 6 min using a microplate reader (Epoch, BioTek Instruments, Minneapolis, MN, USA). The acetylcholinesterase inhibition rate was calculated based on the absorbance data. The AChE inhibition rate is calculated according to Formula (3):

(3) $$Inhibition\%=\frac{K_{E}-K_{S}}{K_{E}}\times 100\%$$

where K_E_ is the initial reaction rate of the enzyme without the inhibition, while K_S_ is the initial reaction rate of the inhibited enzyme.

### 2.6. Anti-α-Glucosidase Capacity Test

The test was conducted according to previous research with minor modifications [7]. In total, 20 μL of ethanolic EO solution, 80 μL of 100 mM PBS (pH 6.8), and 40 μL of α-glucosidase solution (0.25 U/mL) were combined in a microplate, with acarbose serving as the control. The mixture was incubated at 30 °C for 10 min. Subsequently, 20 μL of 3.0 mg/mL 4-nitrophenyl-β-D-glucopyranoside (pNPG) was added, followed by homogenization for 60 s and further incubation at 30 °C for 4 min. Absorbance was measured at 410 nm at 60 s intervals for 6 min. The α-glucosidase inhibition rate is expressed using Equation (3). The IC_50_ value was calculated using nonlinear regression.

### 2.7. Test for β-Lactamase Inhibitory Effect

The β-lactamase inhibitory effect was assessed using a previously described method with some modifications [35]. In total, 20 μL of ethanolic EO solution, 30 μL of 50 mM PBS (pH 7.0), and 100 μL of β-lactamase solution (1000 U/mL) were combined in a microplate, with clavulanate potassium serving as the positive control. The mixture was incubated at 30 °C for 10 min. Subsequently, 50 μL of 0.1 mg/mL nitrocefin was added, followed by an additional 10 min incubation at 30 °C. Absorbance was measured at 489 nm. The β-lactamase inhibition rate was calculated using the following Formula (4):

(4) $$Inhibition\%=\frac{1-{(A}_{S}-A_{sb})}{A_{E}-A_{B}}\times 100\%$$

where A_S_ is the absorbance of the EO-containing sample, A_sb_ is the absorbance of the sample blank reaction, A_E_ is the absorbance of the reaction in which the enzyme was not inhibited, and A_B_ is the absorbance of the blank response. IC_50_ was assessed using nonlinear regression.

### 2.8. Molecular Docking

In the molecular docking experiments, complexes of AChE (*Tetronarce californica*, PDB code: 1EA5), α-glucosidase (*Saccharomyces cerevisiae*, PDB code: 3AJ7), and β-lactamase (*Enterobacter cloacae*, PDB code: 7TI1) were collected from the PDB database. The receptor structures were prepared by removing bound water molecules and ligands using PyMol v2.2.0, and hydrogen atoms were subsequently added to ensure proper protonation states. The 3D structures of the major components of *A. schmidtiana* EO, used as ligands for interaction with the enzyme receptors, were retrieved from the CAS SciFinder Discovery Platform (https://scifinder-n.cas.org/, accessed on 10 March 2025) with energy minimized using Chem3D.

Autodock v4.2.6 was employed to perform semi-flexible molecular docking. Each ligand was subjected to at least 50 runs. Results were analyzed using the Lamarckian genetic algorithm. Binding energies and interactions between the ligands and proteins were evaluated during the docking process. Discovery Studio visualizer and PyMol v2.2.0 were used to visualize the docking results.

## 3. Results and Discussion

### 3.1. EO Yield and Chemical Composition Analysis

**Figure 1:**
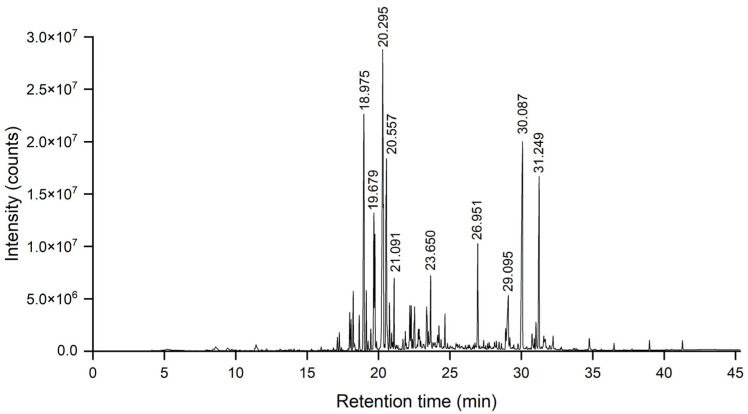
The total ion chromatogram of *A. schmidtiana* EO.

The *A. schmidtiana* EO, obtained through hydrodistillation of 0.14 kg of plant material with 3.0 L ultrapure water, yielded 0.20 mL of red, oily liquid, corresponding to an EO yield of 0.14% (*v*/*w*). The production of plant EO is influenced by numerous factors, including genetic variability, geographical distribution, climatic conditions, seasonal variations, and post-harvest processing, such as drying and storage [36]. The EO yield of A. schmidtiana (0.14%) was comparable to other *Artemisia* species (0.26–0.31%) [37,38,39], indicating normal production levels for this genus. The total ion chromatogram (TIC) of *A. schmidtiana* is shown in Figure 1.

**Figure 2:**
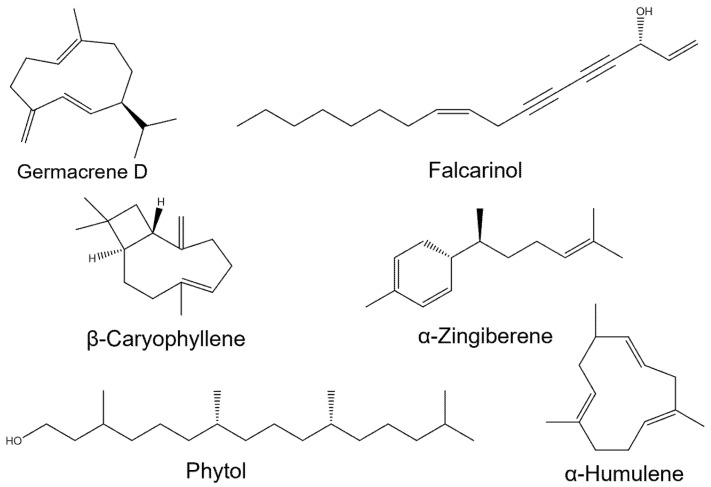
The major compounds in *A. schmidtiana* EO.

EO is a complex mixture of volatile molecules, typically composed of multiple compounds in varying proportions, each exhibiting distinct chemical structures and functionalities. According to elution order, retention time (RT), and RI, each EO component was identified, as listed in Table 1. A total of 80 compounds, amounting to 98.47%, were identified. The major constituents included germacrene D (16.29%), falcarinol (11.02%), β-caryophyllene (9.43%), α-zingiberene (7.93%), phytol (6.06%), α-humulene (4.04%), *n*-hexadecanoic acid (3.38%), hexahydrofarnesyl acetone (3.31%), sesquisabinene (3.29%), and neointermedeol (2.08%). Further classification revealed a sesquiterpenoid-dominated profile (65.87%), followed by aliphatic compounds (18.97%), diterpenoids (6.21%), and monoterpenoids (0.73%) (Figure 2).

Germacrene D (16.29%), the most abundant compound in the EO, primarily exists as (+) and/or (−) enantiomers in EO and is recognized as a significant aromatic compound in the fragrance industry, serving as an antibiotic, repellent, attractant, or pheromone [40,41]. The second most abundant compound, falcarinol (11.02%), has been shown to reduce the expression of the apoptosis marker caspase-3, decrease basal DNA strand breaks, and promote CaCo-2 cell proliferation, thereby preventing and ameliorating colon cancer progression [42]. Falcarinol also exhibits cytotoxicity against human tumor cell lines and exerts kinetic effects on the proliferation of primary mammalian cells [43]. Another notable compound, β-caryophyllene (9.43%), has also garnered attention as a potential broad-spectrum pharmacological agent [44]. β-Caryophyllene extracted from *Aquilaria crassna* EO has been demonstrated to possess remarkable anticancer, antioxidant, and antimicrobial properties [45]. While these major constituents play pivotal roles in the biological activities of *A. schmidtiana* EO, minor components also contribute synergistically or antagonistically, collectively defining the bioactivity of the EO system. Given the chemical diversity of the EO system, we further evaluated its multifaceted biological activities, including antioxidant activity and inhibition of key therapeutic target enzymes associated with diseases.

### 3.2. Antioxidant Activity Analysis of A. schmidtiana EO

Due to the variability among antioxidant testing systems, results from a single assay cannot fully capture the antioxidant potential of samples. The chemical complexity of EO, comprising diverse constituents with unique behaviors, polarities, and functional groups, often leads to inconsistent outcomes depending on the assay used [46]. Therefore, a multi-assay approach is recommended for accurate evaluation.

**Figure 3:**
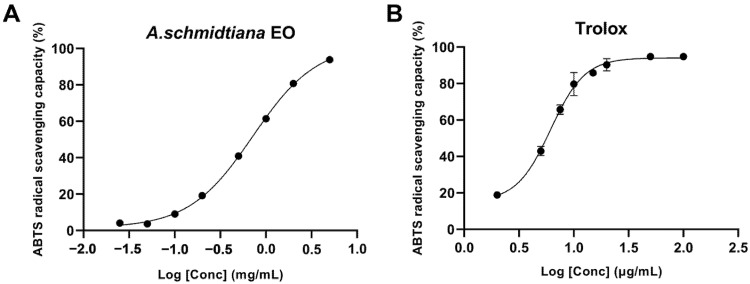
Variation in ABTS radical scavenging percentage with varying concentrations for *A. schmidtiana* EO (**A**) and Trolox (**B**).

The experimental results of DPPH radical scavenging activity are presented in Table 2. At the maximum tested concentration of 5.0 mg/mL, the inhibition rate was 44.9%, while the control Trolox achieved nearly 100% inhibition at the same concentration. Figure 3 shows the S-shaped curve of ABTS radical scavenging activity, which increases with concentration. From the graph, the IC_50_ of the EO was determined to be 0.72 ± 0.02 mg/mL. The EO generally demonstrated potent ABTS radical scavenging activity, significantly outperforming its DPPH radical scavenging capacity.

Previous studies have reported better coefficient of determination (R^2^) and coefficient of variation (CV) values for ABTS assays than DPPH assays, potentially offering a plausible explanation for our results. These differences may arise from the steric hindrance caused by the three phenyl groups surrounding the nitrogen atom in the DPPH radical, which impedes hydrogen atom transfer, or the higher sensitivity of DPPH radicals to reaction conditions, which contributes to the greater accuracy of ABTS assays [47]. Additionally, the stereoselectivity of active compounds and their solubility in various assay systems are considered plausible factors influencing the radical scavenging capacity of EO [48]. Typically, the major terpene compounds in *A. schmidtiana* EO lack polar groups, making it challenging to provide hydrogen atoms and limiting their solubility in DPPH assay media. In contrast, ABTS and FRAP assays rely on electron transfer, making them more effective in detecting the antioxidant capacity of both hydrophilic and lipophilic compounds [49]. Therefore, we further conducted FRAP assays to assess antioxidant properties comprehensively and corroborate the above findings.

Unlike free radical scavenging assays, the FRAP test measures the total reducing capacity of *A. schmidtiana* EO. Iron salts serve as the basis of the FRAP assay, acting as oxidants during electron transfer processes. The reducing capacity of the EO may serve as a reliable predictor of its potential antioxidant activity [50]. In this study, the EO exhibited a FRAP value of 126.61 ± 0.59 μmol/g.

By examining the structures of the major compounds in *A. schmidtiana* EO (Figure 2), it is evident that germacrene D (16.29%), falcarinol (11.02%), and α-zingiberene (7.93%) possess extensive conjugated systems, a common feature among most antioxidants. High conjugation allows electron-donating groups (EDGs) to delocalize additional electrons across overlapping π-bonds, enhancing the ability to quench free radicals [51]. Consequently, stronger conjugation correlates with greater antioxidant capacity. In addition to these major compounds, minor constituents like fokienol (1.46%) and isogermacrene D (0.51%) exhibit significant conjugation, contributing substantially to the EO’s antioxidant activity. Therefore, we hypothesize that the robust antioxidant activity of *A. schmidtiana* EO primarily stems from its abundance of conjugated terpenoids.

### 3.3. Anti-AChE Activity of A. schmidtiana EO

AD, the most prevalent form of dementia, is characterized by oxidative stress and progressive neuronal degeneration, often accompanied by visible Aβ deposits in the brain and reduced acetylcholine levels [52]. A typical therapeutic approach for AD management involves the use of AChE inhibitors, such as galantamine [53]. In this study, *A. schmidtiana* EO demonstrated an AChE inhibition rate of 16.9% at the highest tested concentration of 250 μg/mL. In contrast, the positive control galantamine achieved nearly 100% inhibition at the same concentration (Table 3).

**Figure 4:**
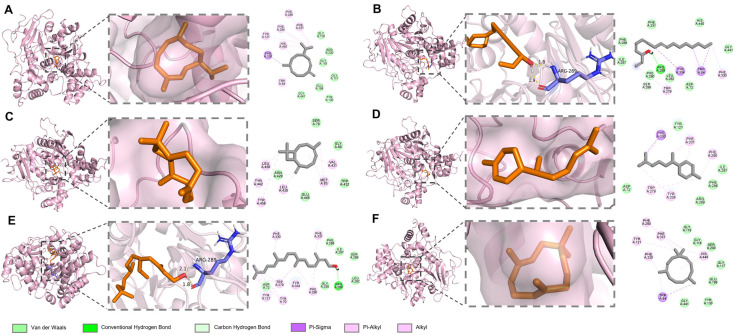
Docking interactions between major compounds and AChE. (**A**) Germacrene D; (**B**) falcarinol; (**C**) β-caryophyllene; (**D**) α-zingiberene; (**E**) phytol; (**F**) α-humulene. From left to right, the overall 3D, partial 3D, and 2D images are presented in order. In 3D images, pink cartoons for AChE protein, orange sticks for small molecules, and purple sticks for amino acid residues bonded with small molecules. In 2D images, green dashed lines are for hydrogen bonding, and pink and purple dashed lines are for hydrophobic interactions.

Molecular docking has become indispensable in recent research for demonstrating the binding affinity of bioactive molecules to target enzymes and validating their potential inhibitory effects. It is reported that small molecules and proteins may exhibit bioactivity when their binding affinity is less than −5.0 kcal/mol, and stable binding occurs when the affinity is below −7.0 kcal/mol [54]. All major compounds docked with AChE exhibited binding energies below −5 kcal/mol, indicating favorable interactions (Table 4). Among these, β-caryophyllene (−7.24 kcal/mol) showed the lowest binding energy, followed by α-humulene (−7.23 kcal/mol) and germacrene D (−7.12 kcal/mol). Notably, these sesquiterpenes lack polar groups capable of forming hydrogen bonds with AChE, suggesting their superior binding may be attributed to significant hydrophobic interactions. Hydrogen bonds are crucial for the stability of protein–ligand complexes, enhancing the stability and longevity of molecules docked in the enzyme’s active site [55]. Alcohol compounds, such as falcarinol and phytol, formed two hydrogen bonds with ARG A: 289, respectively, underscoring their potential inhibitory effects (Figure 4).

Miyazawa et al. revealed that alkenes exhibit more vigorous AChE inhibitory activity than alcohols and ketones. Double bonds in alkenes significantly enhance their inhibitory effect, with allyl-methyl-containing compounds showing potent inhibition. In contrast, oxygen-containing functional groups tend to reduce AChE inhibition [56]. In vivo studies further demonstrated that germacrene D inhibits AChE activity by over 50% in rat brain structures [57]. Moreover, β-caryophyllene has been utilized in treating neurodegenerative diseases [57]. While these compounds demonstrate significant AChE inhibitory activity, the in vitro assay revealed that the EO exhibited only moderate AChE inhibition. This apparent discrepancy may arise from antagonistic interactions among the volatile constituents within the complex EO system, potentially attenuating the collective inhibitory effect [58].

### 3.4. Anti-α-Glucosidase Activity of A. schmidtiana EO

**Figure 5:**
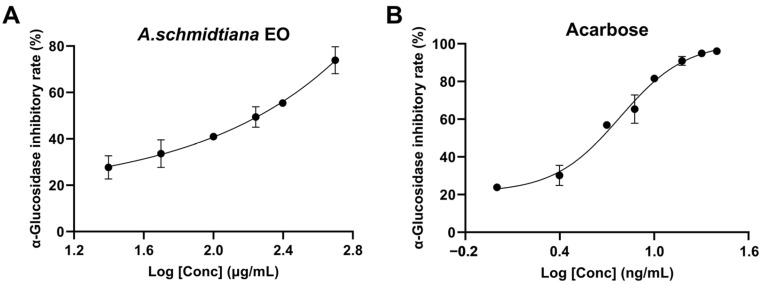
Variation in α-glucosidase inhibitory rate (%) with varying concentrations for *A. schmidtiana* EO (**A**) and acarbose (**B**).

α-Glucosidase, a critical digestive enzyme, accelerates the breakdown of polysaccharides, mainly starch, into glucose by cleaving (1 → 4) glycosidic bonds, thereby elevating blood glucose levels. Inhibitors of α-glucosidase can effectively mitigate postprandial hyperglycemia, potentially relieving diabetes symptoms [59]. In this study, *A. schmidtiana* EO demonstrated an IC_50_ value of 178.80 ± 17.02 μg/mL for α-glucosidase inhibition, and acarbose exhibited an IC_50_ value of 6.04 ± 0.39 ng/mL (Figure 5).

**Figure 6:**
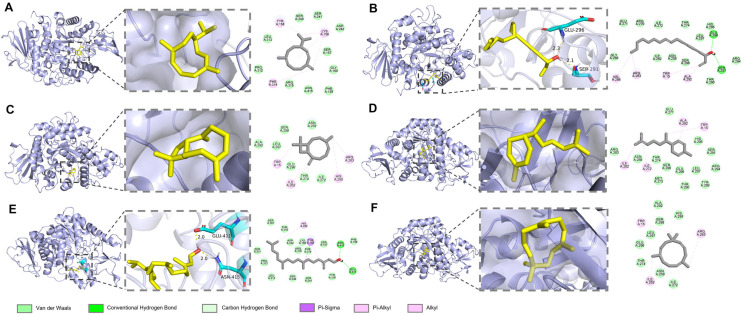
Docking interactions between major compounds and α-glucosidase. (**A**) Germacrene D; (**B)** falcarinol; (**C**) β-caryophyllene; (**D**) α-zingiberene; (**E**) phytol; (**F**) α-humulene. From left to right, the overall 3D, partial 3D, and 2D images are presented in order. In 3D images, purple cartoons for α-glucosidase protein, yellow sticks for small molecules, and blue sticks for amino acid residues bonded with small molecules. In 2D images, green dashed lines are for hydrogen bonding, and pink and purple dashed lines are for hydrophobic interactions.

To further elucidate the mechanism underlying the anti-α-glucosidase activity of *A. schmidtiana* EO, molecular docking was employed to evaluate the interactions between main compounds and the catalytic site of α-glucosidase. As shown in Table 5, the binding energies of all compounds ranged from −5.53 kcal/mol to −6.65 kcal/mol, with no unfavorable interactions observed (Table 5). Similar to the results with AChE, sesquiterpenes did not form hydrogen bonds with α-glucosidase. In contrast, falcarinol formed two hydrogen bonds with protein residues GLU A: 296 and SER A: 291, while phytol established hydrogen bonds with ASN A: 415 and GLU A: 411 (Figure 6). These compounds, characterized by multiple rotatable bonds and higher polarity, demonstrated potential as α-glucosidase inhibitors. β-Caryophyllene (−6.65 kcal/mol) exhibited the lowest binding energy among all compounds, followed closely by germacrene D (−6.59 kcal/mol), α-humulene (−6.46 kcal/mol), and α-zingiberene (−6.23 kcal/mol), likely due to their analogous cycloalkene structures.

Studies have shown that EO rich in terpenes can inhibit key enzymes associated with diabetes, particularly α-glucosidase. Majouli et al. reported that terpenes administered to alloxan-induced diabetic rats exhibited hypoglycemic effects and high antioxidant activity [60]. Currently, several *Artemisia* species, including *A*. *anethifolia*, *A*. *desertorum*, *A*. *latifolia*, *A*. *umbrosa*, *A*. *tanacetifolia*, *A*. *palustris*, *A*. *leucophylla*, and *A*. *commutata*, have been demonstrated to significantly inhibit α-glucosidase [61], highlighting *Artemisia* species’ potential in diabetes treatment.

### 3.5. Anti-β-Lactamase Activity of A. schmidtiana EO

**Figure 7:**
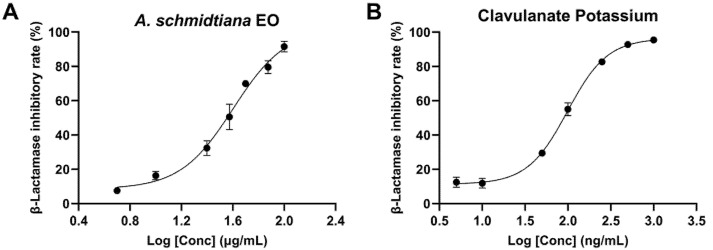
Variation in β-lactamase inhibitory rate (%) with varying concentrations for *A. schmidtiana* EO (**A**) and clavulanate potassium (**B**).

Since the introduction of β-lactam antibiotics, bacterial resistance to these drugs has become an increasingly critical issue. The production of β-lactamases by bacteria represents the most common resistance mechanism to β-lactam antibiotics. A key strategy to combat antibiotic resistance involves using β-lactamase inhibitors [62]. In this study, we evaluated the β-lactamase inhibitory activity of *A. schmidtiana* EO, which exhibited an IC_50_ value of 40.06 ± 8.22 μg/mL. At the same time, the positive control clavulanate potassium demonstrated an IC_50_ of 98.93 ± 3.93 ng/mL (Figure 7).

**Figure 8:**
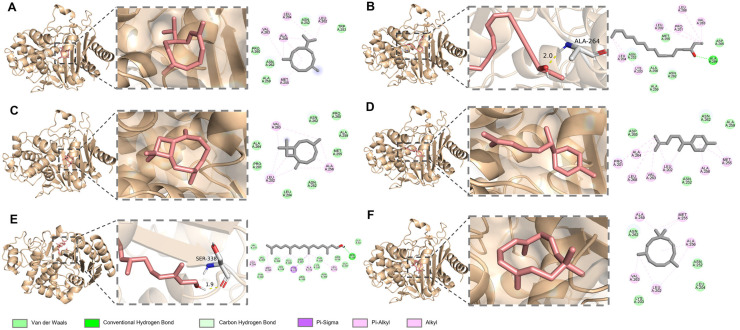
Docking interactions between major compounds and α-glucosidase. (**A**) Germacrene D; (**B**) falcarinol; (**C**) β-caryophyllene; (**D**) α-zingiberene; (**E**) phytol; (**F**) α-humulene. From left to right, the overall 3D, partial 3D, and 2D images are presented in order. In 3D images, wheat cartoons for α-glucosidase protein, salmon sticks for small molecules, and gray sticks for amino acid residues bonded with small molecules. In 2D images, green dashed lines are for hydrogen bonding, and pink and purple dashed lines are for hydrophobic interactions.

Molecular docking analysis revealed detailed interactions between six major components of *A. schmidtiana* EO and the β-lactamase target. The binding energies of these six small molecules with β-lactamase ranged from −5.15 kcal/mol to −6.71 kcal/mol, with no unfavorable interactions observed (Table 6). Falcarinol formed one hydrogen bond with ALA A:264, while phytol established a hydrogen bond with SER A:338 (Figure 8). Although these hydrogen bonds stabilized the binding between the small molecules and the receptor, their relatively high binding energies and inhibition constants suggested limited inhibitory efficacy. Notably, germacrene D (−6.71 kcal/mol) exhibited the lowest binding energy, followed by β-caryophyllene (−6.56 kcal/mol), α-humulene (−6.54 kcal/mol), and α-zingiberene (−6.51 kcal/mol). These compounds primarily interacted with β-lactamase through van der Waals forces, potentially contributing to their inhibitory effects. In addition, minor constituents present in EO may also play a critical role in inhibiting β-lactamase activity.

## 4. Conclusions

In this study, the chemical composition, antioxidant activity, anti-AChE, anti-α-glucosidase, and anti-β-lactamase activities of *A. schmidtiana* EO were investigated for the first time. A total of 80 compounds were identified, with the major compounds being germacrene D (16.29%), falcarinol (11.02%), β-caryophyllene (9.43%), α-zingiberene (7.93%), phytol (6.06%), and α-humulene (4.04%). The EO exhibited antioxidant activity and inhibitory effects on AChE, α-glucosidase, and β-lactamase in vitro, suggesting its potential as a source of enzyme inhibitors. Molecular docking studies further supported the in vitro findings. These bioactivities collectively indicate the pharmacological potential of *A. schmidtiana* EO. However, the in vitro results provide only preliminary data on its biological activities. Future in vivo studies are essential to evaluate potential side effects, therapeutic efficacy, and targeted delivery of the EO. Therefore, integrating in vivo research with pharmacodynamic and pharmacokinetic analyses is crucial for further developing and applying *A. schmidtiana* EO.

## Figures and Tables

**Table 1:** Chemical composition of EO distilled from *A. schmidtiana*.

No.	RT	Compound *	RI^calc^	RI^lib^	Area (%)	CAS ID
1	8.588	7-Octenal	999	996	0.45%	21573-31-9
2	9.417	Limonene	1029	1029	0.27%	5989-27-5
3	11.425	Linalool	1103	1099	0.46%	78-70-6
4	15.980	Bornyl acetate	1288	1285	0.11%	76-49-3
5	17.120	δ-EIemene	1339	1338	0.38%	20307-84-0
6	17.256	Silphinene	1345	1345	0.48%	74284-57-4
7	17.982	α-Copaene	1378	1376	1.02%	3856-25-5
8	18.075	Modephene	1382	1385	0.85%	68269-87-4
9	18.227	α-Isocomene	1389	1386	1.57%	65372-78-3
10	18.298	β-Cubebene	1392	1389	0.18%	13744-15-5
11	18.342	(+)-Sativen	1394	1396	0.11%	3650-28-0
12	18.653	β-Isocomene	1409	1412	0.93%	71596-72-0
13	18.975	β-Caryophyllene	1424	1419	9.43%	87-44-5
14	19.144	β-Copaene	1432	1432	1.39%	18252-44-3
15	19.264	α-Bergamotene	1438	1435	0.23%	17699-05-7
16	19.46	Isogermacrene D	1448	1448	0.51%	317819-80-0
17	19.679	α-Humulene	1458	1454	4.04%	6753-98-6
18	19.749	Sesquisabinene	1462	1462	3.29%	58319-04-3
19	19.864	(*Z*)-Muurola-4(15),5-diene	1467	1463	0.22%	157477-72-0
20	20.295	Germacrene D	1488	1481	16.29%	23986-74-5
21	20.415	1-Pentadecene	1494	1492	0.12%	13360-61-7
22	20.557	α-Zingiberene	1501	1495	7.93%	495-60-3
23	20.639	α-Muurolene	1505	1499	0.24%	10208-80-7
24	20.770	β-Bisabolene	1511	1509	1.19%	495-61-4
25	20.917	γ-Cadinene	1519	1513	0.42%	39029-41-9
26	21.091	Cadina-1(10),4-diene	1528	1526	1.68%	16729-01-4
27	21.239	(*E*)-γ-Bisabolene	1535	1533	0.12%	53585-13-0
28	21.713	α-Agarofuran	1560	1550	0.24%	5956-12-7
29	21.877	(*E*)-Nerolidol	1568	1564	0.36%	40716-66-3
30	21.932	1,5-Epoxysalvial-4-ene	1571	1573	0.12%	88395-47-5
31	22.199	Spathulenol	1584	1576	1.30%	6750-60-3
32	22.292	Caryophyllene oxide	1589	1581	1.08%	1139-30-6
33	22.384	Longifolenaldehyde	1594	1591	0.18%	19890-84-7
34	22.526	Fokienol	1601	1596	1.46%	33440-00-5
35	22.788	Humulene oxide II	1615	1606	0.45%	19888-34-7
36	22.843	β-Biotol	1618	1612	0.43%	19902-26-2
37	22.892	9-Cedranone	1621	1617	0.23%	13567-40-3
38	22.957	Junenol	1624	1620	0.15%	472-07-1
39	23.383	Cedrelanol	1647	1640	1.80%	5937-11-1
40	23.497	Humulenol	1653	1650	0.49%	19888-00-7
41	23.574	α-Cadinol	1657	1653	0.13%	481-34-5
42	23.650	Neointermedeol	1662	1660	2.08%	5945-72-2
43	23.814	Aromadendrene oxide-(1)	1670	1672	0.23%	85710-39-0
44	23.961	Aromadendrene oxide-(2)	1678	1678	0.17%	-
45	24.146	Eudesma-4(15),7-dien-1β-ol	1688	1688	0.53%	119120-23-9
46	24.234	4(15),5,10(14)-Germacratrien-1-ol	1693	1690	0.62%	81968-62-9
47	24.381	Acorenone B	1701	1701	0.24%	21653-33-8
48	24.654	1-Pentadecanal	1716	1715	0.84%	2765-11-9
49	24.828	(*E*)-Farnesol	1726	1722	0.13%	106-28-5
50	25.434	Xanthorrhizol	1760	1752	0.18%	30199-26-9
51	25.521	β-Bisabolenal	1765	1768	0.17%	147029-14-9
52	25.647	Costol	1773	1776	0.20%	515-20-8
53	25.783	*n*-Pentadecanol	1780	1778	0.12%	629-76-5
54	26.094	β-Bisabolenol	1798	1790	0.10%	147126-90-7
55	26.280	(8*R*,8a*S*)-3,4,6,7,8,8a-Hexahydro-5-methyl-8-(1-methylethyl)-2-naphthalenemethanol	1809	1803	0.11%	135118-52-4
56	26.372	Eudesm-11-en-4-α,6-α-diol	1814	1808	0.11%	405554-95-2
57	26.951	Hexahydrofarnesyl acetone	1849	1848	3.31%	502-69-2
58	27.365	Diisobutyl phthalate	1873	1869	0.21%	84-69-5
59	27.643	(8*Z*,11*Z*)-Heptadecadienal	1890	1886	0.14%	56797-42-3
60	27.747	Methyl 4,7,10-hexadecatrienoate	1896	1892	0.15%	17364-31-7
61	28.254	Methyl palmitate	1927	1926	0.23%	112-39-0
62	28.445	Verrucarol	1939	1939	0.20%	2198-92-7
63	28.609	Isophytol	1949	1948	0.15%	505-32-8
64	28.904	Dibutyl phthalate	1967	1965	0.54%	84-74-2
65	29.095	*n*-Hexadecanoic acid	1979	1968	3.38%	57-10-3
66	29.771	Octadecanal	2022	2021	0.21%	638-66-4
67	30.087	Falcarinol	2043	2038	11.02%	21852-80-2
68	30.757	1-Octadecanol	2086	2082	0.56%	112-92-5
69	30.911	Methyl linoleate	2096	2092	0.26%	112-63-0
70	31.026	Methyl linolenate	2104	2099	0.93%	301-00-8
71	31.249	Phytol	2119	2114	6.06%	150-86-7
72	31.577	Linoleic acid	2141	2132	0.47%	60-33-3
73	31.631	Oleic acid	2145	2141	0.26%	112-80-1
74	31.680	Gamolenic acid	2148	2144	0.35%	506-26-3
75	31.980	Octadecanoic acid	2169	2172	0.17%	57-11-4
76	32.226	(8*E*)-8-Octadecenyl acetate	2185	2189	0.50%	2195-90-6
77	34.763	Octadecanamide	2367	2374	0.50%	124-26-5
78	36.492	Pentacosane	2497	2500	0.18%	629-99-2
79	38.969	Heptacosane	2698	2700	0.26%	593-49-7
80	41.282	Nonacosane	2898	2900	0.27%	630-02-4
	Monoterpenoids				0.73%	
	Sesquiterpenoids				65.87%	
	Diterpenoids				6.21%	
	Aliphatic compounds				18.97%	
	Other compounds				6.69%	
	Total identified				98.47%	

* All compounds were identified by linear retention indices (LRIs) and mass spectrometry (MS). % Peak areas were calculated from the total ion chromatogram; RI^Calc^: calculated retention index; RI^lib^: retention index was obtained from the NIST/EPA/NIH 2023 Mass Spectral Database. LRI: relative retention indices calculated against *n*-alkanes; identification method based on the RRI of authentic compounds on the HP-5MS column; MS, identified based on computer matching of the mass spectra with NIST/EPA/NIH 2023 Mass Spectral Database and comparison with the literature data.

**Table 2:** Antioxidant activities expressed as IC_50_ values for DPPH and ABTS, and the antioxidant capacity of FRAP assays.

Tested Samples	DPPH (IC_50_)	ABTS (IC_50_)	FRAP Antioxidant Capacity
*A. schmidtiana* EO	>5 mg/mL (44.9%)	0.72 ± 0.02 mg/mL	126.61 ± 0.59 μmol/g
Trolox	9.93 ±0.29 µg/mL	6.15 ± 0.23 µg/mL	-

**Table 3:** Enzyme inhibitory activities expressed as IC_50_ values for anti-acetylcholinesterase, anti-α-glucosidase, and anti-β-lactamase assays.

Tested Samples	AChE (IC_50_)	α-Glucosidase (IC_50_)	β-Lactamase (IC_50_)
*A. schmidtiana* EO	>250 μg/mL (16.9%)	178.8 ± 17.02 μg/mL	40.06 ± 8.22 μg/mL
Galantamine	130.0 ± 2.0 ng/mL	-	-
Acarbose	-	6.04 ± 0.39 ng/mL	-
Clavulanate Potassium	-	-	98.93 ± 3.93 ng/mL

**Table 4:** The best docking results of AChE with major compounds of *A. schmidtiana* EO.

Components	Binding Energy (kcal/mol)	Van der Waals Hydrogen Bond Desolvation Energy (kcal/mol)	Ligand Efficiency (kcal/mol)	Inhibition Constant (μmol/L)
Germacrene D	−7.12	−7.43	−0.47	6.04
Falcarinol	−6.50	−9.93	−0.36	17.33
β-Caryophyllene	−7.24	−7.26	−0.48	4.93
α-Zingiberene	−6.67	−7.85	−0.44	12.97
Phytol	−6.58	−10.70	−0.31	14.91
α-Humulene	−7.23	−7.23	−0.48	5.04

**Table 5:** The best docking results of α-glucosidase with major compounds of *A. schmidtiana* EO.

Components	Binding Energy (kcal/mol)	Van der Waals Hydrogen Bond Desolvation Energy (kcal/mol)	Ligand Efficiency (kcal/mol)	Inhibition Constant (μmol/L)
Germacrene D	−6.59	−6.88	−0.44	14.79
Falcarinol	−5.97	−9.51	−0.33	42.25
β-Caryophyllene	−6.65	−6.65	−0.44	13.4
α-Zingiberene	−6.23	−7.43	−0.42	27.22
Phytol	−5.53	−9.55	−0.26	87.87
α-Humulene	−6.46	−6.47	−0.43	18.55

**Table 6:** The best docking results of β-lactamase with major compounds of *A. schmidtiana* EO.

Components	Binding Energy (kcal/mol)	Van der Waals Hydrogen Bond Desolvation Energy (kcal/mol)	Ligand Efficiency (kcal/mol)	Inhibition Constant (μmol/L)
Germacrene D	−6.71	−7.01	−0.45	12.00
Falcarinol	−5.48	−8.97	−0.30	96.02
β-Caryophyllene	−6.56	−6.55	−0.44	15.64
α-Zingiberene	−6.51	−7.69	−0.43	16.99
Phytol	−5.19	−9.18	−0.25	157.06
α-Humulene	−6.54	−6.54	−0.44	16.05

## Data Availability

The data presented in this study are available from the corresponding author upon request.

## Abbreviations

The following abbreviations are used in this manuscript:

ABTS	2,2-azino-bis (3-ethylbenzothiazoline-6-sulfonic acid)
ACh	Acetylcholine
AChE	Acetylcholinesterase
AD	Alzheimer’s disease
*A. schmidtiana*	*Artemisia schmidtiana* Maxim.
ATCI	Acetylthiocholine iodide
Aβ	Amyloid-β
CV	Coefficient of variation
DM	Diabetes mellitus
DPPH	2,2-diphenyl-1-picrylhydrazyl
DTNB	5,5′-dithiobis-(2-nitrobenzoic acid)
EDGs	Electron-donating groups
EI	Electron ionization
EO	Essential oil
FRAP	Ferric-reducing antioxidant power
GC-FID	Gas chromatography with flame ionization detection
GC-MS	Gas chromatography–mass spectrometry
LRI	Linear retention indices
PBS	Potassium phosphate buffer solution
pNPG	4-nitrophenyl-β-D-glucopyranoside
RI	Retention index
RT	Retention time
R^2^	Coefficient of determination
TEAC	Trolox equivalent antioxidant concentration
TIC	Total ion chromatogram
TPTZ	2,4,6-tripyridyl-s-triazine
Trolox	6-hydroxy-2,5,7,8-tetramethylchroman-2-carboxylic acid
